# Reliability and usability of a portable spirometer compared to a laboratory spirometer

**DOI:** 10.1186/s12890-025-03690-1

**Published:** 2025-05-10

**Authors:** Yi Gao, Binmiao Liang, Xibin Su, Wentao Rao, Huitong Cheng, Chonghui Fan, Xinxin Yu, Yanqing Xie, Beilan Shen, Jing Du, Linwei Li, Binjian Liu

**Affiliations:** 1 National Center for Respiratory Medicine, State Key Laboratory of Respiratory Disease, National Clinical Research Center for Respiratory Disease, Guangzhou Institute of Respiratory Health, The First Affiliated Hospital of Guangzhou Medical University, Guangzhou, Guangdong People’s Republic of China; 2 Department of Pulmonary and Critical Care Medicine, West China Hospital of Sichuan University, Chengdu, Sichuan People’s Republic of China; 3 Department of Clinical Medicine, Medcaptain Medical Technology Co., Ltd, 7F, Building 7A, Phase 3 of Shenzhen International Innovation Valley, Liuxin Fourth Street, Nanshan District, Shenzhen, Guangdong People’s Republic of China; 4 Anesthesia and Respiratory Division, Medcaptain Medical Technology Co., Ltd., Shenzhen, Guangdong People’s Republic of China

**Keywords:** Portable spirometer, Pulmonary function test, Spirometry, Chronic obstructive pulmonary disease, Asthma

## Abstract

**Background:**

Access to spirometry remains limited due to the expense and inconvenience of stationary laboratory spirometers, which may compromise the diagnosis and management of chronic respiratory diseases (CRDs), such as chronic obstructive pulmonary disease (COPD) and asthma. Portable spirometers offer potential advantages over laboratory spirometers in terms of affordability, user-friendliness, and portability. The objective of this study is to evaluate the reliability and usability of a portable spirometer (Medcaptain VC-30 Pro) compared to a conventional laboratory spirometer (Jaeger MasterScreen PFT).

**Methods:**

In this multi-center, randomized, open-label crossover study, 132 subjects from two hospitals were recruited to perform pulmonary function tests using both the portable spirometer and the laboratory spirometer. Forced expiratory volume in one second (FEV_1_), forced vital capacity (FVC), FEV_1_/FVC ratio, peak expiratory flow (PEF), forced expiratory flow between 25 and 75% of FVC (FEF_25-75%_), vital capacity (VC), maximal voluntary ventilation (MVV), and forced expiratory volume in six seconds (FEV_6_) were compared for correlation and agreement between two spirometers. The concordance of their spirometric abnormality diagnoses and severity classifications was assessed. An additional 30 healthy volunteers were recruited to perform a pulmonary function test by themselves after a session guided by specialists to evaluate the usability of the portable spirometer.

**Results:**

A total of 126 recruited participants achieved acceptable pulmonary function test results. The intraclass correlation coefficients (ICCs) for primary outcomes FEV_1_ and FVC were 0.994 and 0.993, respectively (both *p* < 0.001). ICCs for other outcomes ranged from 0.968 to 0.995 (all *p* < 0.001). The Bland–Altman analysis showed that FEV_1_ and FVC met preset acceptable criteria, with 96.0% of values falling within the 95% limits of agreement (LoA). Cohen’s kappa statistics for the diagnosis of spirometric abnormality and classification of severity were 0.872 and 0.878, respectively. In the usability test, 28 out of 30 volunteers obtained a Grade A result.

**Conclusions:**

The portable spirometer exhibited a strong correlation and agreement with a high-quality laboratory spirometer, as well as concordance in spirometric abnormality diagnosis and severity classification. Non-specialist can obtain acceptable results using this portable spirometer.

**Supplementary Information:**

The online version contains supplementary material available at 10.1186/s12890-025-03690-1.

## Background

Chronic respiratory diseases (CRDs) rank as the third leading cause of mortality worldwide, with chronic obstructive pulmonary disease (COPD) causing the most deaths and asthma having the highest prevalence [1]. Spirometry is considered the gold standard for diagnosing and monitoring CRDs [2]. It assesses lung function parameters, such as forced expiratory volume in one second (FEV_1_) and forced vital capacity (FVC), to identify airflow limitation indicative of obstructive lung disease and other abnormalities [3]. International guidelines recommend the regular use of spirometry for diagnosis and management in asthma and COPD [4, 5]. However, access to spirometry is limited, especially in primary care settings and low- or middle-income countries [6–9].

Traditional laboratory spirometers are expensive, bulky, and require regular calibration and trained personnel for operation, leading to potential underdiagnosis or overdiagnosis of respiratory conditions [10, 11]. The lack of spirometry utilization also increases the risk of severe asthma or COPD exacerbations [12]. The coronavirus disease 2019 (COVID-19) pandemic further impeded diagnostic capabilities due to the closure of pulmonary function testing laboratories to reduce transmission risks, compromising the quality of care for many patients with CRDs [13].

In light of these challenges, portable spirometers offer significant advantages, including affordability, user-friendliness, and portability. Studies have demonstrated that some portable spirometers exhibit good consistency with laboratory spirometers [14–18]. However, systematic differences in lung function values may exist between portable and laboratory spirometers, emphasizing the need to evaluate their accuracy before implementation in primary care settings [19]. Furthermore, both tests were performed by qualified pulmonary function specialists, raising the question of whether individuals lacking experience in pulmonary function testing, such as primary care physicians and patients themselves, can effectively conduct spirometry tests and obtain high-quality results.

This multi-center randomized study aimed to assess the reliability of spirometry measurements obtained through a portable spirometer (Medcaptain VC-30 Pro) compared to a conventional laboratory spirometer (Jaeger MasterScreen PFT). Additionally, the usability of the portable device was evaluated among volunteers inexperienced in pulmonary function tests. Demonstrating the portable spirometer’s reliability and usability could provide substantial support for its implementation to improve access to respiratory disease screening and management, particularly in primary care and home settings.

## Methods

### Study design and participants

This was a multi-center, randomized, open-label crossover validation study conducted at two distinguished medical institutions in the People’s Republic of China: The First Affiliated Hospital of Guangzhou Medical University in Guangdong Province and West China Hospital of Sichuan University in Sichuan Province. Subjects aged 4 years and older were randomly recruited during routine clinic visits and underwent spirometry tests using both spirometers. During enrollment, the ratio of participants diagnosed with COPD or asthma was required to be no less than 30%. Exclusion criteria followed the guidelines outlined by the American Thoracic Society/European Respiratory Society (ATS/ERS) for spirometry contraindications [10]. Prior to enrollment and randomization, written informed consent was obtained from all participants or their legal guardians.

The study adhered to the principles set forth in the Declaration of Helsinki and received approval from the Ethics Committee of The First Affiliated Hospital of Guangzhou Medical University (2022 No. 165, November 8, 2022) and the Ethics Committee of West China Hospital of Sichuan University (2023 No. 145, January 28, 2023).

### Randomization and intervention

A stratified block randomization method was used to allocate participants to two study groups. Randomization was performed using SAS software (SAS Institute Inc., USA). A predefined seed and block size were utilized to generate random group assignments, ensuring a 1:1 ratio between the two groups across both centers. This process yielded a randomization schedule for all participants, assigning each a unique subject number and initial testing device (portable or laboratory spirometer). Randomization cards were created with concealed group allocations. Upon enrollment and informed consent, investigators revealed the allocation by removing the concealment on the card and proceeded with the designated intervention. All participants performed FVC, slow vital capacity (SVC), and maximal voluntary ventilation (MVV) tests under the guidance of a certified pulmonary function specialist at a consistent location. Each specialist was a physician with substantial clinical experience in conducting spirometry tests and had prior experience with both the portable and laboratory spirometers used in the study. To ensure accuracy and consistency, each participant was required to perform a minimum of three technically acceptable maneuvers that met repeatability criteria for FEV_1_ and FVC, as recommended by the Standardization of Spirometry 2019 Update by ATS/ERS [10]. Following a rest period of 10 min, participants repeated the spirometry test using the other device.

The portable spirometer used in the study was the Medcaptain VC-30 Pro (Medcaptain Medical Technology Co., Ltd., PRC). This handheld spirometer consists of a differential pressure and flow sensor, a cylindrically shaped handle, and a Bluetooth module for wireless connectivity to a PC or tablet running the associated application. The application displays real-time flow-volume and volume-time curves, with spirometry values calculated from the flow data. The laboratory spirometer employed was the Jaeger MasterScreen PFT (CareFusion, USA), which is widely recognized as one of the most prevalent pulmonary function systems. This stationary, computer-based system incorporates a pneumotachograph flow sensor. Testing is conducted with the participant connected to the device via a disposable mouthpiece and nose clip. All tests were performed with participants in a seated position.

### Parameters

For each spirometer, the following pulmonary function parameters were recorded: FEV_1_, FVC, FEV_1_/FVC ratio, peak expiratory flow (PEF), forced expiratory flow between 25 and 75% of FVC (FEF_25-75%_), vital capacity (VC), and MVV. FEV_1_ and FVC were designated as the primary outcomes of this study, while the secondary outcomes comprised PEF, FEF_25-75%_, MVV, and VC. Since studies have shown that forced expiratory volume in six seconds (FEV_6_) is more reproducible than FVC, and that FEV_1_/FEV_6_ may have higher sensitivity than FEV_1_/FVC for detecting early airflow limitation, we also performed a post-hoc analysis of FEV_6_ and FEV_1_/FEV_6_ as exploratory outcomes at one center [10]. The most optimal spirometry values were extracted for further analysis.

### Sample size

The sample size was determined based on the need to detect a minimal clinically important difference of 0.2 L in FEV_1_ and FVC between the devices. The standard deviation was estimated to be 0.7 L, with a desired power of 80% and a significance level (alpha) of 0.05. Consequently, a minimum sample size of 99 patients was calculated. Taking into account the potential for data ineligibility and missing values, the study aimed to enroll 132 participants, with an equal distribution of 66 participants at each center.

### Statistical analysis

Data were analyzed using Python 3.10 (Python Software Foundation, USA). Continuous variables were reported as mean and standard deviation or median and interquartile range, as appropriate. Categorical variables were reported as frequency and percentage.

The primary analysis involved a comparison between portable and laboratory spirometer readings. Paired Student’s t-tests or Wilcoxon rank-sum tests were employed, depending on the distribution of the data. To evaluate agreement between the devices, Pearson correlation coefficients, intraclass correlation coefficients (ICCs), and Bland–Altman plots were utilized. The interpretation of Pearson correlation coefficients and ICCs followed previously reported classification standards, where a value greater than 0.9 indicates excellent correlation [20, 21]. The acceptable deviation from the reference reading was set at within ± 3% or ± 0.050 L for FEV_1_ and FVC measurements [22]. The acceptable 95% limits of agreement (LoA) were preliminarily set as ± 0.35 L for FEV_1_ and ± 0.5 L for FVC, with no less than 95% of the differences in FEV_1_ and FVC falling within these limits [23].

Based on the pulmonary function test results, participants were categorized into four pattern groups: (1) normal, characterized by an FEV_1_/FVC ratio of ≥ 0.7 and FVC ≥ 80% of the predicted value; (2) restrictive, characterized by an FEV_1_/FVC ratio of ≥ 0.7 and FVC < 80% of the predicted value; (3) obstructive defect, characterized by an FEV_1_/FVC ratio of < 0.7 and FVC ≥ 80% of the predicted value; and (4) mixed, characterized by an FEV_1_/FVC ratio of < 0.7 and FVC < 80% of the predicted value [3]. Furthermore, the severity of spirometric abnormality was graded according to ATS/ERS guidelines: mild (FEV_1_ > 70% of the predicted value), moderate (FEV_1_ between 60 − 69% of the predicted value), moderately severe (FEV_1_ between 50 − 59% of the predicted value), severe (FEV_1_ between 35 − 49% of the predicted value), and very severe (FEV_1_ < 35% of the predicted value) [3, 24]. Specificity and sensitivity for the diagnosis were calculated, and Cohen’s Kappa coefficient was employed to evaluate the level of diagnostic agreement, with interpretations as follows: almost perfect (> 0.90), strong (0.80 − 0.90), moderate (0.60 − 0.79), weak (0.40 − 0.59), minimal (0.21 − 0.39), and none (0 − 0.20) [25].

Subgroup analyses were performed based on disease types to assess the consistency of agreement between the two spirometers.

Statistical significance was determined using a two-sided p-value threshold of < 0.05. Missing data were not imputed, and a per-protocol analysis was conducted, considering only participants with complete and eligible portable and laboratory spirometry data in accordance with ATS/ERS standards [10].

### Usability sub-study

To assess the usability of the portable spirometer, a convenience sample of 30 healthy adult volunteers was recruited, following the recommended sample size for a pilot study [26]. Initially, the volunteers underwent their first pulmonary function test using the portable spirometer under the guidance of a specialist and achieved acceptable results. The following day, the volunteers were provided with a video tutorial from the manufacturer and a quality control checklist to independently perform a maximum of eight spirometry maneuvers. The device’s software included built-in quality feedback and direction to assist the users, with no additional coaching provided.

A trained respiratory therapist, unaware of the volunteers’ identities, evaluated the quality of the unsupervised results. The maneuvers were graded automatically by the device according to the criteria established by ATS [10, 27]. The number of successful tests, defined as three maneuvers meeting the repeatability criteria and deemed acceptable (Grade A), was calculated. Detailed criteria for repeatability and acceptability were presented in Supplemental Table 1. The unsupervised results were then compared to those obtained under specialist supervision. Usability was defined as the volunteers’ ability to independently produce acceptable and reproducible spirometry results compared to tests conducted under professional guidance.

## Results

### Demographic and clinical characteristics

**Fig. 1:**
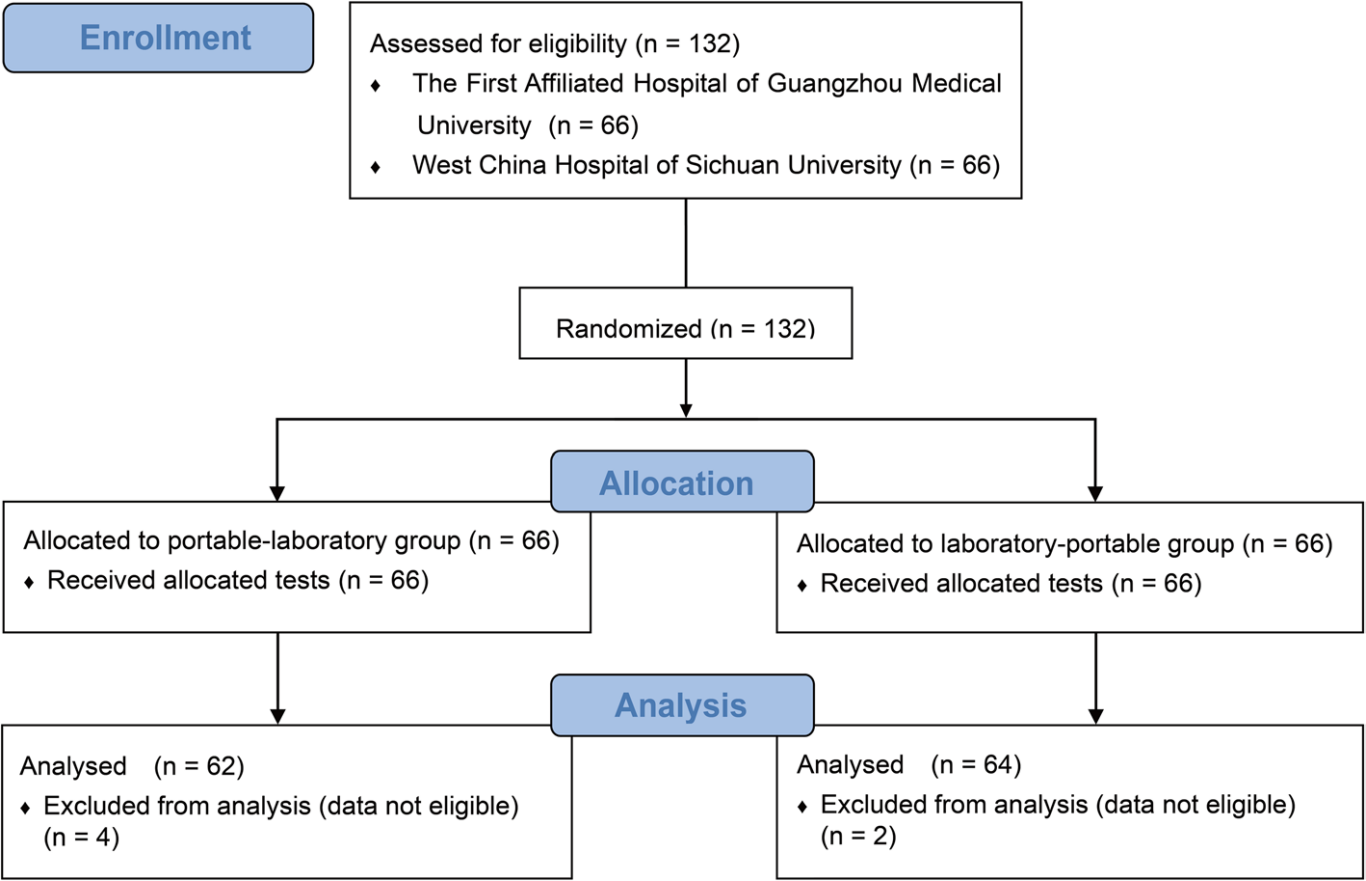
Consolidated Standards of Reporting Trials (CONSORT) participant flow diagram

A total of 132 participants were recruited and randomly assigned to two groups: one group performed the pulmonary function test using the laboratory spirometer first, while the other group used the portable spirometer first (Fig. 1). Out of the recruited participants, 126 successfully completed testing using both devices and obtained data that adhered to ATS/ERS criteria. However, there were 8 participants for whom predicted values were missing. Consequently, these participants were excluded from the respective analyses.

The baseline demographic and clinical characteristics of the participants are summarized in Table 1. Overall, the study population covered a wide age range and a variety of respiratory diagnoses, with a balanced representation of genders and a mostly normal body mass index (BMI) distribution. Regarding respiratory diagnoses, 19.0% of participants had COPD, 23.0% had asthma, 7.9% had other CRDs, while the remaining 50.0% did not have any specific lung disease.

**Table 1:** Basic information of per-protocol participants (*N* = 126)

Characteristic	Mean ± SD or *n* (%)
Age (years)	40.3 ± 20.5
< 20	26 (20.6)
20 − 40	34 (27.0)
40 − 60	39 (31.0)
≥ 60	27 (21.4)
Gender	
Male	68 (54.0)
Female	58 (46.0)
Height (cm)	157.8 ± 13.0
Weight (kg)	57.6 ± 15.5
BMI (kg/m^2^)	22.7 ± 4.4
< 18.5	21 (16.7)
18.5–25	71 (56.3)
≥ 25	34 (27.0)
Diagnosis	
COPD	24 (19.0)
Asthma	29 (23.0)
Other CRDs	10 (7.9)
No specific lung diseases	63 (50.0)

*BMI* Body mass index, *COPD* Chronic obstructive pulmonary disease, *CRDs* Chronic respiratory diseases

### Primary and secondary outcome comparisons between devices

Mean values for primary and secondary outcomes are presented in Table 2. For FEV_1_, the mean value with the portable spirometer was 2.40 ± 0.78 L, compared to 2.41 ± 0.79 L with the laboratory spirometer. For FVC, the portable spirometer had a mean of 3.28 ± 0.94 L versus 3.27 ± 0.94 L for the laboratory spirometer. For secondary outcomes, PEF, FEF_25−75%_ and MVV showed systematic differences between the two spirometers, with p-values less than 0.05. The remaining outcomes of FEV_1_/FVC, VC, FEV_6_, and FEV_1_/FEV_6_ showed no statistically significant differences between the two devices.

**Table 2:** Mean values for primary and secondary outcomes in participants

Parameter	Mean ± SD		*p*-value
	VC-30 Pro	MasterScreen PFT	
FEV_1_	2.40 ± 0.78	2.41 ± 0.79	0.050
FVC	3.28 ± 0.94	3.27 ± 0.94	0.583
FEV_1_/FVC	75.03 ± 15.74	75.41 ± 15.05	0.171
PEF	6.10 ± 2.03	6.32 ± 2.10	< 0.001
FEF_25**−**75%_	2.25 ± 1.28	2.08 ± 1.24	< 0.001
VC	3.30 ± 0.95	3.29 ± 0.95	0.523
MVV	92.61 ± 33.62	93.81 ± 33.82	0.034
FEV_6_	3.14 ± 1.06	3.11 ± 1.08	0.178
FEV_1_/FEV_6_	68.93 ± 13.16	69.63 ± 13.23	0.193

*FEV*_*1*_ Forced expiratory volume in 1 s, *FVC* Forced vital capacity, *PEF* Peak expiratory flow, *FEF*_*25−75%*_ Forced expiratory flow between 25 and 75% of FVC, *VC* Vital capacity, *MVV* Maximal voluntary ventilation, *FEV*_*6*_ Forced expiratory volume in 6 s

### Correlation and agreement between devices

**Fig. 2:**
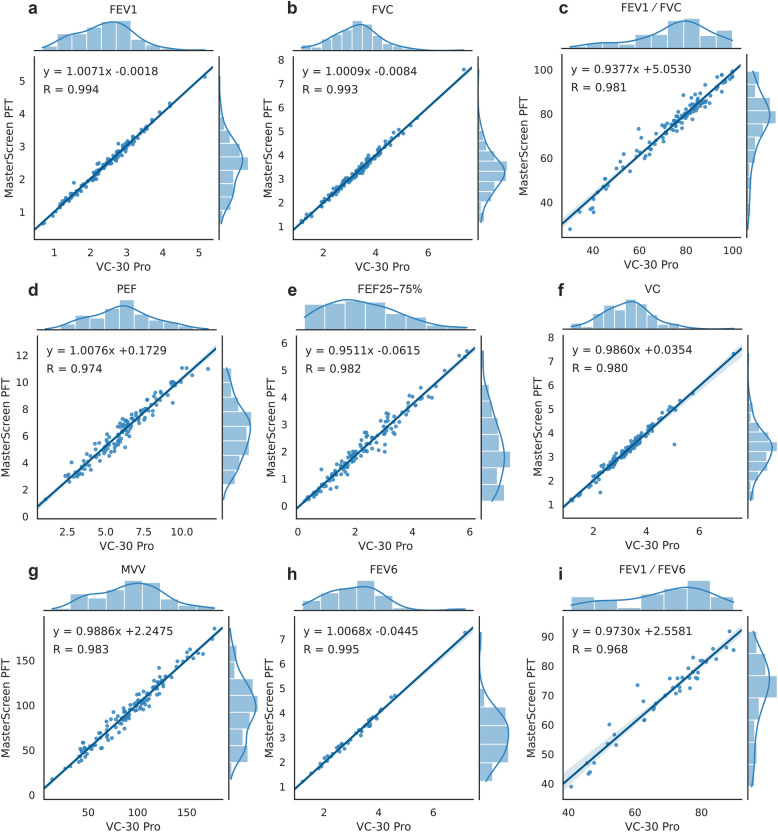
Distribution and correlation of spirometry results obtained from the portable spirometer (VC-30 Pro) and laboratory spirometer (MasterScreen PFT)

Scatter plots, Pearson correlation coefficients, ICCs and 95% LoA for primary and secondary outcomes between the two spirometers are shown in Fig. 2 and Table 3. There was excellent correlation between the portable and laboratory spirometer for both primary outcomes. The Pearson correlation coefficient R was 0.994 for FEV_1_ and 0.993 for FVC (both p < 0.001). The ICCs were 0.994 for FEV_1_ and 0.993 for FVC, indicating excellent reliability.

**Table 3:** Correlation and agreement of primary and secondary outcomes between the portable and laboratory spirometers

Parameter	R	ICC	Mean difference (95% LoA)	Points within 95% LoA (%)
FEV_1_	0.994	0.994	-0.015 (-0.185, 0.155)	96.0
FVC	0.993	0.993	0.005 (-0.207, 0.218)	96.0
FEV_1_/FVC	0.981	0.980	-0.380 (-6.456, 5.696)	96.0
PEF	0.974	0.973	-0.219 (-1.149, 0.711)	94.4
FEF_25**−**75%_	0.982	0.981	0.172 (-0.309, 0.652)	92.1
VC	0.980	0.980	0.011 (-0.362, 0.384)	98.4
MVV	0.983	0.983	-1.195 (-13.479, 11.089)	94.4
FEV_6_	0.995	0.995	0.023 (-0.187, 0.233)	97.5
FEV_1_/FEV_6_	0.968	0.968	-0.700 (-7.256, 5.856)	95.0

*FEV*_*1*_ Forced expiratory volume in 1 s, *FVC* Forced vital capacity, *PEF* Peak expiratory flow, *FEF*_*25****−****75%*_ Forced expiratory flow between 25 and 75% of FVC, *VC* Vital capacity, *MVV* Maximal voluntary ventilation, *FEV*_*6*_ Forced expiratory volume in 6 s, *ICC* Intraclass correlation coefficient, *LoA* Limit of agreement

**Fig. 3:**
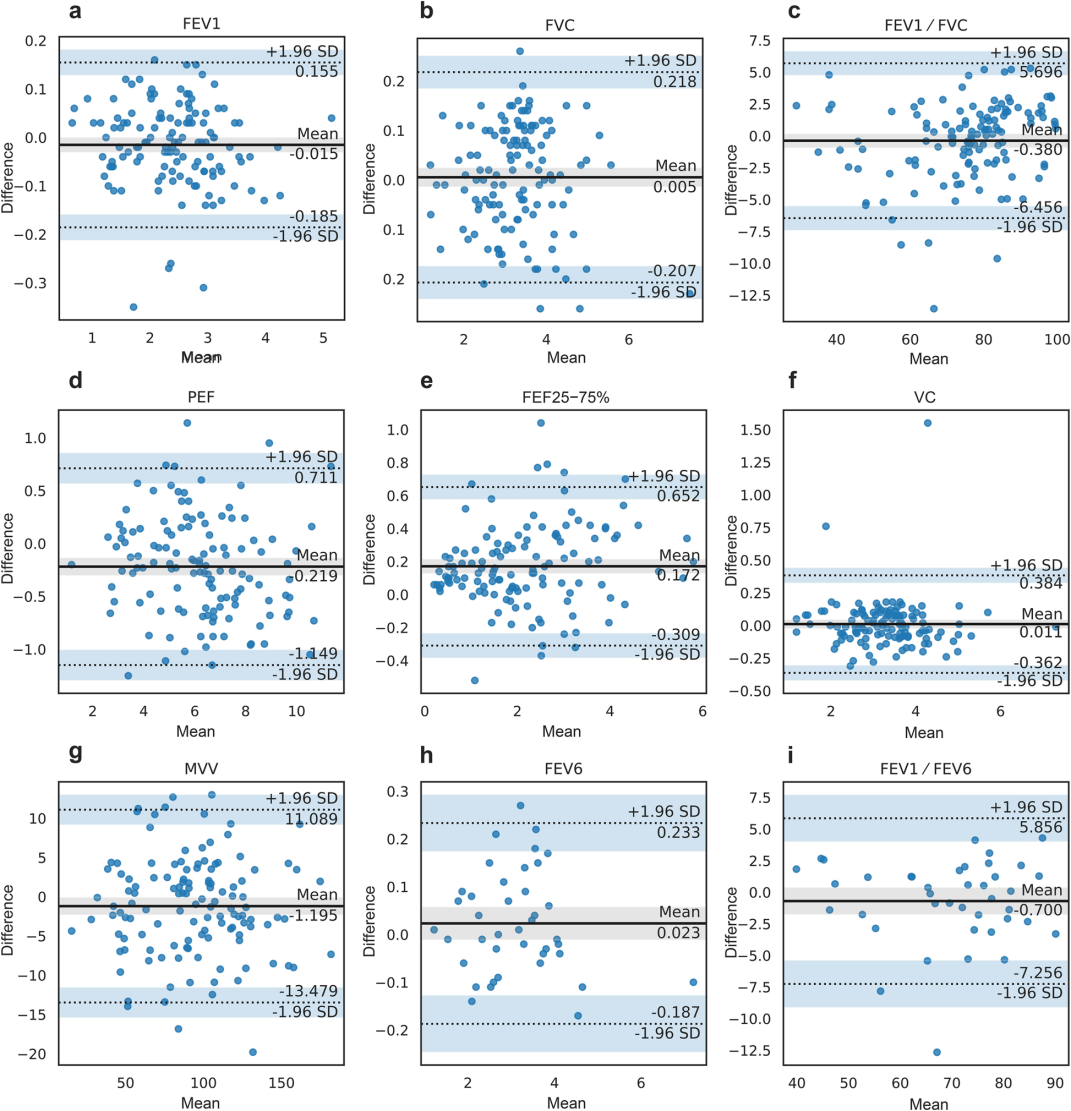
Bland–Altman plots of spirometry results and their 95% LoA

The Bland–Altman plots are shown in Fig. 3. The analysis revealed that 96.0% of FEV_1_ and FVC differences were within the predefined acceptable limits. The 95% LoA were -0.185 to 0.155 L for FEV_1_ and -0.207 to 0.218 L for FVC. Both primary outcomes met preset clinically acceptable criteria.

The remaining outcomes of FEV_1_/FVC, PEF, FEF_25−75%_, VC, MVV, FEV_6_, and FEV_1_/FEV_6_ showed good correlation and agreement between devices with Pearson coefficients ranging from 0.968 to 0.995 and ICCs between 0.968 and 0.995 (Table 3). In the Bland–Altman analysis, over 92% of differences fell within the predefined limits (Fig. 3).

### Concordance of spirometric abnormality and severity classification

Based on the spirometry results, participants were classified as having normal, restrictive, obstructive, or mixed patterns (Table 4). The portable and laboratory spirometers showed strong agreement in identifying abnormal pulmonary function, with a kappa statistic of 0.872. The sensitivity and specificity of the portable spirometer for detecting any abnormality (restrictive, obstructive, or mixed) were 91.4% and 95.2%, respectively. The classification of abnormality severity also demonstrated good concordance between the two devices, with a kappa statistic of 0.878 (Table 5).

**Table 4:** Spirometric classification of pulmonary function abnormality by two spirometers (Sensitivity = 91.4%, specificity = 95.2%, κ = 0.872)

VC-30 Pro	MasterScreen PFT				
	Normal	Obstructive	Restrictive	Mixed	Total
Normal	79	2	1	0	82
Obstructive	3	24	0	0	27
Restrictive	0	0	3	0	3
Mixed	0	1	0	5	6
Total	82	27	4	5	118

**Table 5:** Spirometric classification of severity by two spirometers (κ = 0.878)

VC-30 Pro	MasterScreen PFT				
	Normal	Mild	Moderate to Moderately Severe	Severe to Very Severe	Total
Normal	79	2	1	0	82
Mild	3	24	0	0	27
Moderate to Moderately Severe	0	0	3	0	3
Severe to Very Severe	0	1	0	5	6
Total	82	27	4	5	118

### Subgroup analysis

Subgroup analysis was performed to evaluate the consistency of agreement between the portable and laboratory spirometers. When stratified by disease status, the results remained marginally unchanged across different participant groups (Supplemental Fig. 1 − 4). Among COPD patients, the Pearson correlation coefficients were 0.984 for FEV_1_ and 0.993 for FVC, while the ICC were 0.983 and 0.992, respectively. The 95% LoA were -0.216 to 0.203 L for FEV_1_ and -0.086 to 0.252 L for FVC, with 91.7% of differences falling within these limits. Similarly, for asthma patients, the Pearson correlation coefficients were 0.992 for FEV_1_ and 0.996 for FVC, with ICC values of 0.992 and 0.996, respectively. The 95% LoA were -0.217 to 0.178 L for FEV_1_ and -0.201 to 0.196 L for FVC, with 96.6% and 93.1% of differences within these limits.

### Usability outcomes

A sample of 30 healthy volunteers was recruited to assess the usability of the portable spirometer. Among the participants, 17 (56.7%) were male. The age distribution revealed that 21 individuals (70%) were in the 20 to 29 years age group, while 4 participants (13.3%) fell within the 30 to 39 years age range, and 5 (16.7%) were between 40 and 50 years old. After their initial spirometry test guided by a specialist, 28 (93.3%) of them achieved Grade A test results independently. Only one volunteer from the 20 to 29 age group and one from the 40 to 49 age group obtained a Grade B result (only two acceptable maneuvers but met the repeatability criteria).

**Fig. 4:**
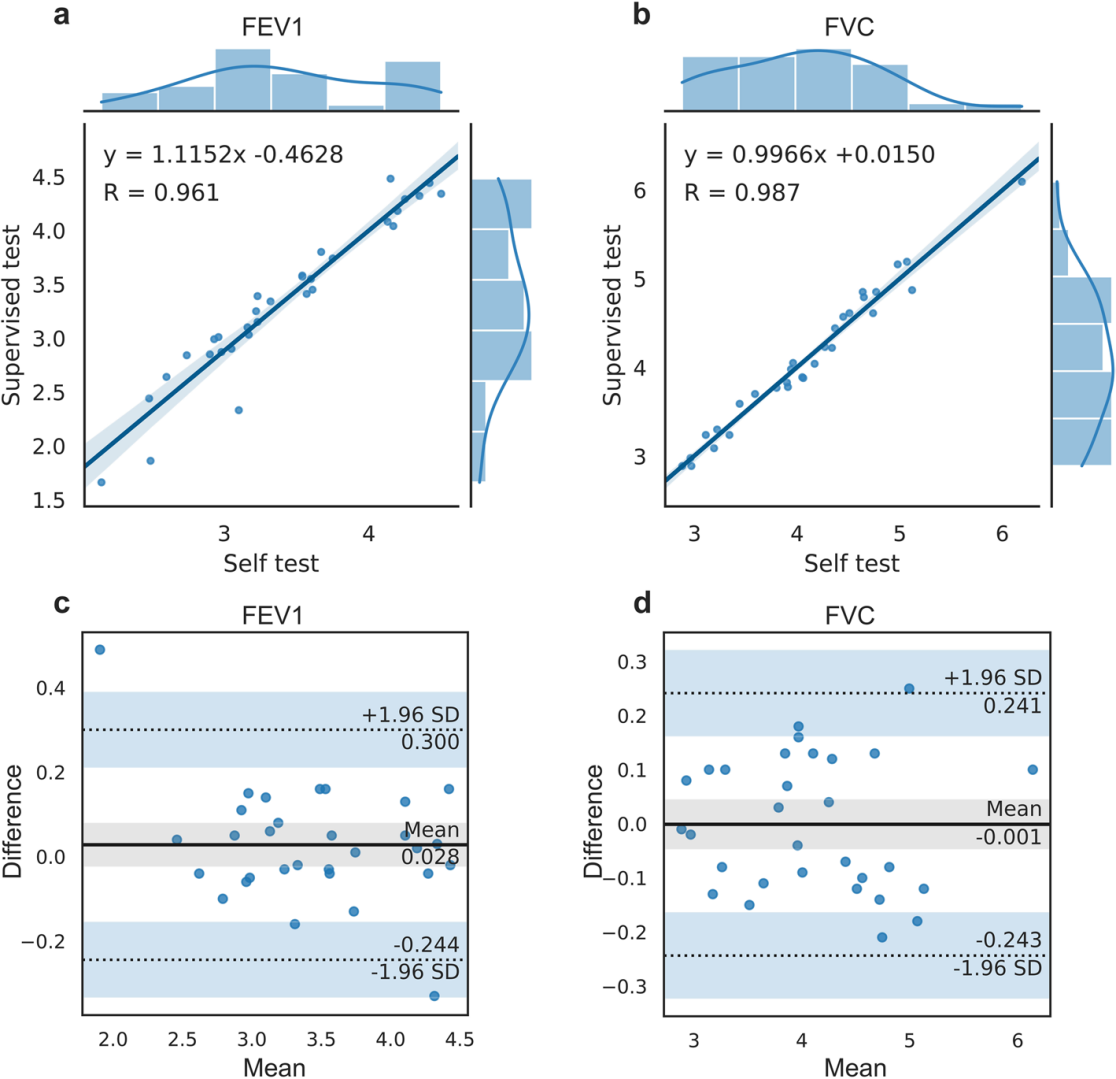
Distribution, correlation and Bland–Altman plots of supervised tests and self-tests spirometry results. **a** distribution and correlation of FEV_1_ results; **b** distribution and correlation of FVC results. **c** Bland–Altman plot of FEV_1_ differences; **d** Bland–Altman plot of FVC differences

The scatter plots and Bland–Altman plots of FEV_1_ and FVC are presented in Fig. 4. The Pearson correlation coefficients for FEV_1_ and FVC were 0.961 and 0.987, and ICCs were 0.950 and 0.987, respectively, indicating a strong association between these variables. The mean difference between supervised tests and self-tests was 0.028 (95% LoA -0.244 to 0.300) for FEV_1_ and -0.001 (95% LoA -0.243 to 0.241) for FVC. In the Bland–Altman analysis, 93.3% of the FEV_1_ differences and 96.7% of the FVC differences fell within the 95% LoA.

## Discussion

In this multi-center, randomized, open-label crossover validation study comparing the portable spirometer Medcaptain VC-30 Pro to the laboratory spirometer Jaeger MasterScreen PFT, the two devices showed excellent agreement in measuring key pulmonary function parameters. The primary outcomes of FEV_1_ and FVC demonstrated strong correlation and acceptable mean differences between the portable and laboratory spirometers, indicating that the portable spirometer is reliable and produces consistent results comparable to the laboratory spirometer for these key parameters. For other parameters, including FEV_1_/FVC, PEF, FEF_25−75%_, VC, MVV, FEV_6_, and FEV_1_/FEV_6_, the portable spirometer provided accurate measurements and exhibited good reliability as well, validating the portable spirometer’s capability to effectively identify and classify respiratory conditions. These results suggest that portable spirometry is a dependable alternative to traditional laboratory spirometry, particularly in diverse clinical settings.

The findings align with previous studies that highlighted the accuracy of portable spirometers, but with narrower 95% LoA for key pulmonary function parameters like FEV_1_ and FVC, demonstrating even better agreement in this study [14–18, 28]. In the COPD and asthma subgroups, the agreements between these two devices remained marginally unchanged. These results provide further evidence to support the applicability of portable spirometers as viable alternatives to conventional stationary pulmonary function testing. Additionally, the analysis in one center showed that FEV_6_ demonstrated good correlation. Since studies have reported that FEV_1_/FEV_6_ is useful for diagnosing airflow obstruction in adults, the reliability of portable spirometers could contribute to further investigations concerning these novel parameters [10].

This study is one of the few to assess SVC and MVV using a portable spirometer. SVC assesses vital capacity by inhaling and exhaling in a deliberate and unhurried manner, as opposed to the forceful maneuver used to determine FVC [29]. This method is particular valuable for elderly individuals who may struggle to execute an FVC maneuver without experiencing coughing episodes [29]. MVV is defined as the subject’s maximum minute volume of ventilation estimated over 12 to 15 s [30]. Factors such as impaired coordination of respiratory muscles, musculoskeletal chest wall disorders, neurological conditions, chronic illness-induced deconditioning, and ventilatory abnormalities can contribute to a decrease in MVV [30]. Although MVV is non-specific, it can be useful in certain circumstances, such as preoperative evaluation and assessing respiratory muscle fatigue [30]. The portable spirometer demonstrated excellent agreement with the laboratory spirometer for both SVC and MVV, further validating its performance across a broader range of pulmonary function tests.

The adoption of portable spirometers presents significant implications for clinical practice and health policy, particularly regarding cost-effectiveness and accessibility [31, 32]. Portable spirometers are more affordable than stationary spirometers, require minimal maintenance, and eliminate the need for dedicated testing space. While this study did not conduct a formal cost-effectiveness analysis, this affordability and convenience of portable devices make them particularly well-suited for resource-limited settings, primary care offices, and even for home monitoring. Enhanced accessibility allows for more frequent testing, which can lead to earlier detection and better management of CRDs.

Given the global burden of CRDs, which account for 4 million deaths in 2019 and affected approximately 454.6 million individuals globally, improving access to diagnostic tools like spirometry is critical [1]. For example, obstructive sleep apnea (OSA), a condition often underdiagnosed due to the high costs and complexity of standard diagnostic methods, could benefit from the increased availability of spirometry [33]. Previous meta-analyses have evaluated several biomarkers to guide OSA clinical decision-making, but further in-depth research is still needed before these biomarkers can be applied in practice [33–36]. Although not a primary diagnostic tool for OSA, spirometer can provide valuable insights into mixed patterns of obstruction and restriction often seen in patients with this condition [37–39]. With regular monitoring, healthcare providers can track disease progression, identify exacerbations, and implement timely interventions. This proactive approach can improve outcomes for patients with conditions such as asthma and COPD by optimizing treatment plans and encouraging better self-management.

In addition to affordability, the usability of portable spirometers is crucial for their successful implementation. However, only a few studies have investigated the usability of portable spirometers, and rather than focusing on the reliability of self-testing, these studies concentrated on user experience [40–42]. Our pilot sub-study provides promising preliminary evidence supporting the feasibility of utilizing portable spirometry as a self-administered test, even by non-specialists. With simple guidance, such as tutorial videos and checklists, most volunteers achieved reliable spirometry results. This is particularly important given that previous studies have highlighted the challenges of meeting spirometry standards in general practice settings [43, 44].

The ability to conduct testing in home settings or primary care environments, rather than relying solely on specialist referrals, can enhance the management of CRDs. However, challenges related to device usability, data accuracy, and patient adherence must be addressed to fully realize this potential. In this context, integrating portable spirometers with telehealth systems represents a significant opportunity to enhance respiratory care. Certain portable devices, like the VC-30 series, can upload pulmonary function data to hospital systems for specialist analysis, allowing for remote monitoring and timely adjustments to treatment plans without in-person visits. During virtual consultations, clinicians can review spirometry results with patients, facilitating immediate discussions about health status. Educational resources and tutorials can support accurate self-testing at home, while integration with electronic health record (EHR) systems enhances care coordination. For infectious respiratory diseases like COVID-19, remote spirometry could enable safe, continuous monitoring of affected individuals without risking exposure to healthcare workers or other patients. Additionally, telemedicine platforms can send alerts for concerning results, promoting timely interventions, and empowering patients to actively manage their respiratory health, ultimately leading to improved outcomes.

The findings of this study have important implications for primary care, where the reliability and usability of portable spirometers enhance diagnostic and monitoring capabilities. These devices allow for accurate spirometry assessments during routine visits, potentially improving the early detection and management of CRDs. Furthermore, the feasibility of patient-administered tests introduces a new model for continuous monitoring of chronic respiratory conditions that could reduce the need for frequent in-person visits. To ensure successful implementation, structured training programs for healthcare providers and patients are essential. The ease of use demonstrated in our usability study suggests that such training could be efficiently integrated into primary care workflows.

This study was strengthened by its multi-center design, randomized order of tests, and an adequate sample size that enabled the detection of clinically significant differences between devices. Additionally, the participants represented a common spectrum of chronic respiratory conditions and age ranges. However, certain limitations must be acknowledged. The lack of z-score comparisons due to limitations in the laboratory spirometers restricts the ability to evaluate deviations from normal ranges. Moreover, the absence of bronchodilator responsiveness testing limits the ability to assess the portable spirometer’s performance in detecting reversible airway obstruction, a key diagnostic feature in asthma. Without post-bronchodilator data, we cannot ascertain whether changes in spirometry measurements are attributable to the devices used or to underlying airway dynamics specific to asthma. Therefore, the portable spirometer’s performance in capturing the variable airflow limitation characteristic of asthma remains unexplored. Finally, the sampling bias present in our pilot sub-study may underestimate the real-world challenges patients encounter when using medical devices unsupervised, and may lead to misinterpretation of the results. Further studies are warranted to comprehensively evaluate the diagnostic performance and usability of portable spirometers in diverse settings.

## Conclusion

The Medcaptain VC-30 Pro portable spirometer exhibited a strong correlation and agreement with a high-quality laboratory spirometer in measuring lung function, as well as concordance in spirometric abnormality diagnosis and severity classification. This portable spirometer offers ease of use, making it suitable for implementation in primary care and home settings. The adoption of portable spirometers of this nature holds promise in enabling early diagnosis and effective management of CRDs.

## Supplementary Information

Supplementary Material 1: Supplemental Figure 1. Distribution and correlation of spirometry results in COPD subgroup.

Supplementary Material 2: Supplemental Figure 2. Bland-Altman plots of spirometry results and their 95% LoA in COPD subgroup.

Supplementary Material 3: Supplemental Figure 3. Distribution and correlation of spirometry results in asthma subgroup.

Supplementary Material 4: Supplemental Figure 4. Bland-Altman plots of spirometry results and their 95% LoA in asthma subgroup.

Supplementary Material 5: Supplemental Table 1. Acceptability and repeatability criteria for maneuvers.

## Data Availability

The datasets generated and/or analysed during the current study are not publicly available to protect participant privacy but are available from the corresponding author upon reasonable request.

## Abbreviations

CRD: Chronic respiratory disease
COPD: Chronic obstructive pulmonary disease
FEV_1_: Forced expiratory volume in one second
FVC: Forced vital capacity
COVID-19: Coronavirus disease 2019
ATS: American Thoracic Society
ERS: European Respiratory Society
SVC: Slow vital capacity
MVV: Maximal voluntary ventilation
PEF: Peak expiratory flow
FEF_25-75%_: Forced expiratory flow between 25 and 75% of FVC
VC: Vital capacity
FEV_6_: Forced expiratory volume in six seconds
ICC: Intraclass correlation coefficient
LoA: Limits of agreement
BMI: Body mass index
OSA: Obstructive sleep apnea
EHR: Electronic health record
