# Supplementary figures and images for: Reliability and usability of a portable spirometer compared to a laboratory spirometer
Source: BMC Pulm Med. 2025 May 10;25:228. doi: 10.1186/s12890-025-03690-1 (PMC12065281; doi:10.1186/s12890-025-03690-1)

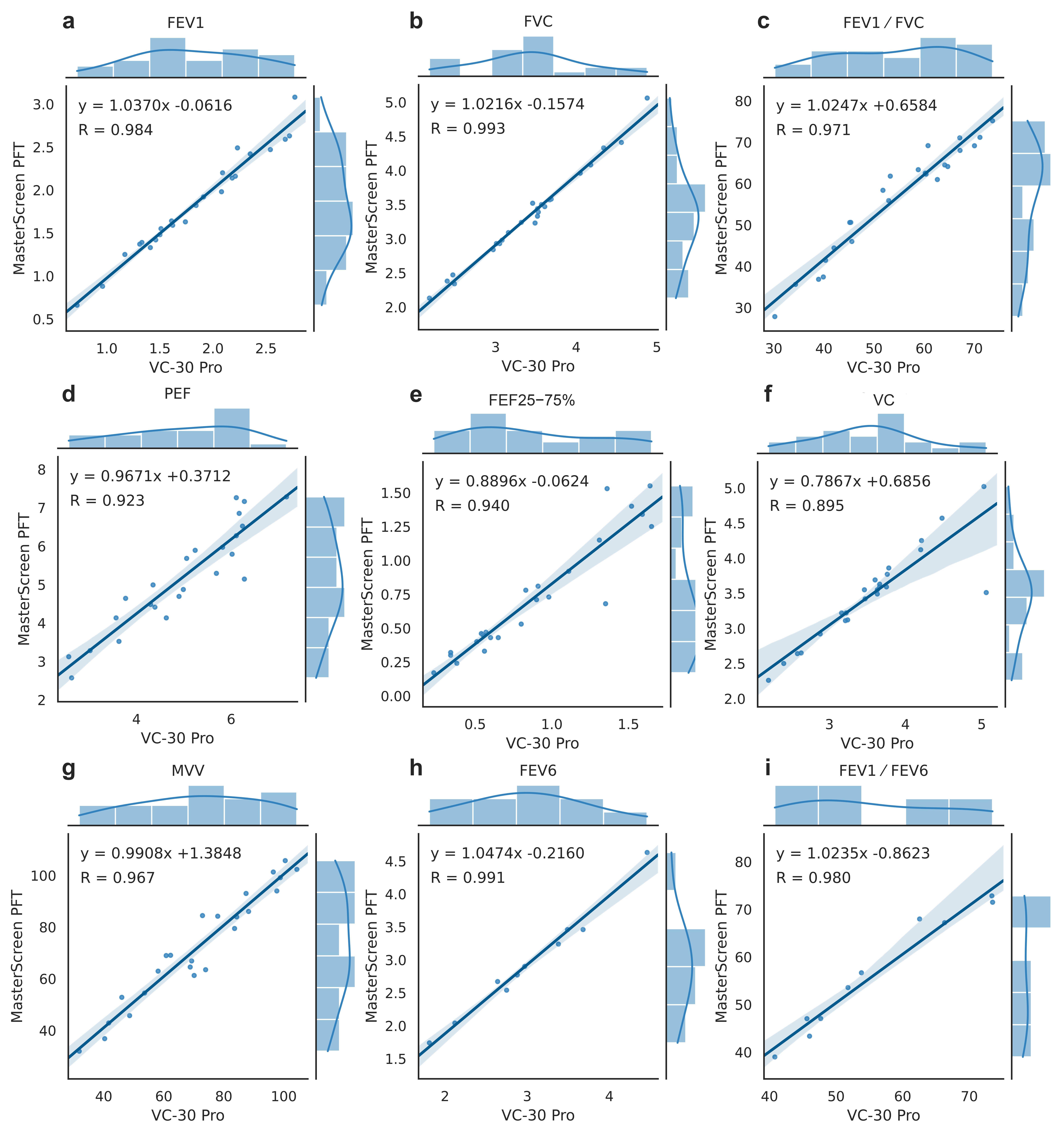

Supplement: Supplementary file 1 — Supplementary Material 1: Supplemental Figure 1. Distribution and correlation of spirometry results in COPD subgroup. [file 12890_2025_3690_MOESM1_ESM.jpg]

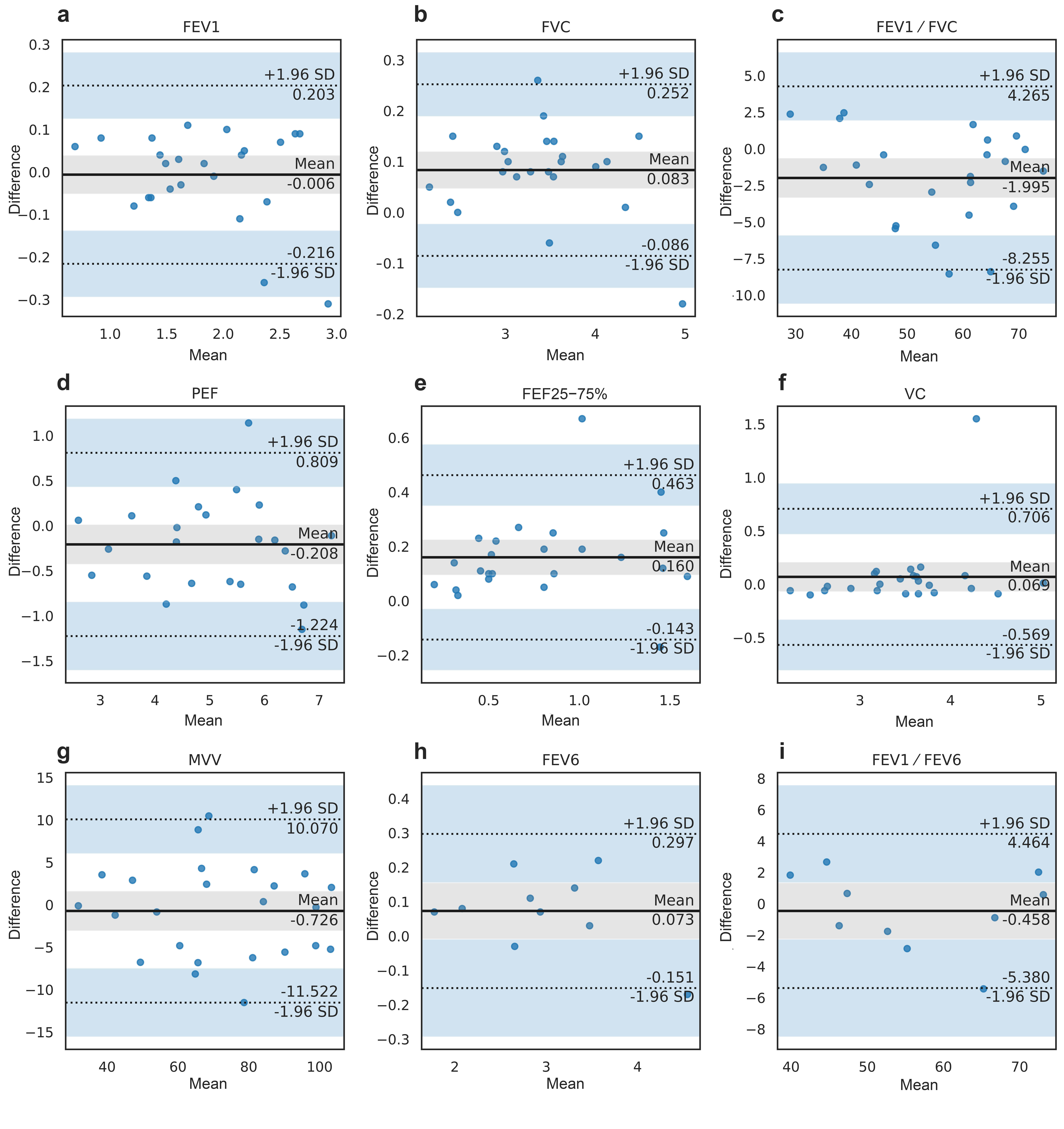

Supplement: Supplementary file 2 — Supplementary Material 2: Supplemental Figure 2. Bland-Altman plots of spirometry results and their 95% LoA in COPD subgroup. [file 12890_2025_3690_MOESM2_ESM.jpg]

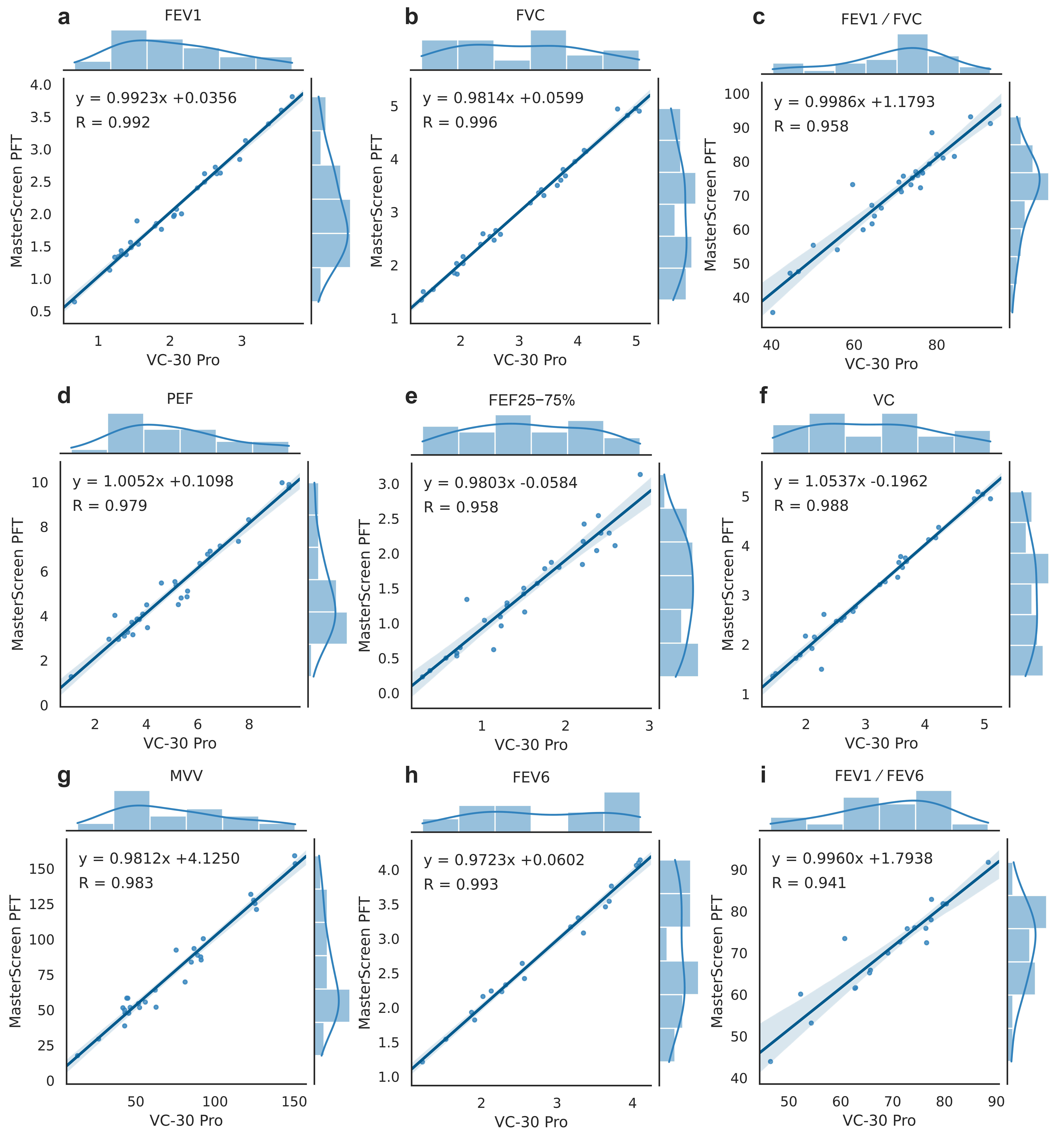

Supplement: Supplementary file 3 — Supplementary Material 3: Supplemental Figure 3. Distribution and correlation of spirometry results in asthma subgroup. [file 12890_2025_3690_MOESM3_ESM.jpg]

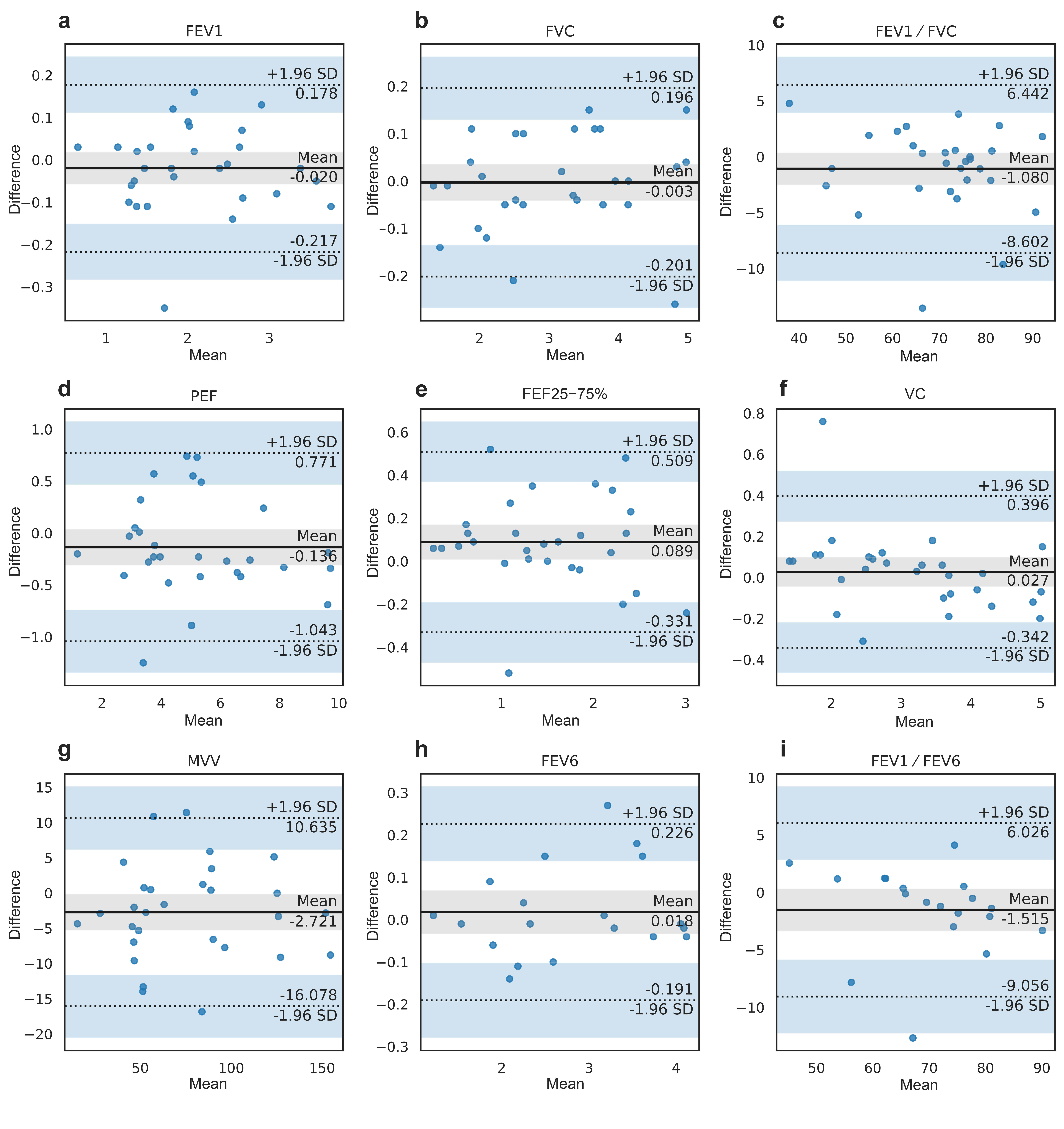

Supplement: Supplementary file 4 — Supplementary Material 4: Supplemental Figure 4. Bland-Altman plots of spirometry results and their 95% LoA in asthma subgroup. [file 12890_2025_3690_MOESM4_ESM.jpg]
