# Supplementary material for: Reliability and usability of a portable spirometer compared to a laboratory spirometer
Source: BMC Pulm Med. 2025 May 10;25:228. doi: 10.1186/s12890-025-03690-1 (PMC12065281; doi:10.1186/s12890-025-03690-1)
Supplement: Supplementary file 5 — Supplementary Material 5: Supplemental Table 1. Acceptability and repeatability criteria for maneuvers. [file 12890_2025_3690_MOESM5_ESM.docx]

**Supplementary Table 1. Acceptability and repeatability criteria for maneuvers**

| **Criterion** |
| --- |
| **A maneuver is considered acceptable if** |
| It is free from artefacts: |
| Cough during the first second of exhalation |
| Glottis closure that influences the measurement |
| Early termination or cut-off |
| Effort that is not maximal throughout |
| Leak |
| Obstructed mouthpiece |
| It has good starts: |
| Extrapolated volume < 5% of FVC or 0.15 L, whichever is greater |
| It shows satisfactory exhalation: |
| Duration of ≥ 6s (3s for children) or plateau in the volume-time curve or  If the subject cannot or should not continue to exhale |
| **Maneuvers are considered repeatable if** |
| The two largest values of FVC are within 0.150 L of each other |
| The two largest values of FEV_1_ are within 0.150 L of each other |

FVC: forced vital capacity; FEV_1_: forced expiratory volume in one second. Adapted from Miller et al (1).

**References:**

1. Miller MR, Hankinson J, Brusasco V, Burgos F, Casaburi R, Coates A, et al. Standardisation of spirometry. Eur Respir J. 2005;26(2):319-38.
